# Recurrent retroperitoneal hemorrhage in a patient with tuberous sclerosis complex: a case report

**DOI:** 10.1186/1757-1626-1-424

**Published:** 2008-12-30

**Authors:** Massimo Chiarugi, Maria Carola Martino, Marsia Pucciarelli, Luigi Decanini, Claudio Vignali

**Affiliations:** 1 Department of Surgery, University of Pisa, Pisa, PI, Italy; 2 Department of Oncology, Transplantation and New Technologies, University of Pisa, Pisa, PI, Italy; 3 Department of Surgery, University of Pisa, Santa Chiara Hospital, 67, Via Roma, 56100 Pisa, PI, Italy

## Abstract

**Background:**

Tuberous sclerosis complex (TSC) is an autosomal dominant disorder. It is characterized by seizures, mental retardation and hamartomatous lesions, including facial angiofibroma, subependymal giant cell astrocytoma, cardiac rhabdomyoma and renal angiomyolipoma (AML). AMLs can bleed severely in the retroperitoneal space.

**Case presentation:**

Herein, we present the case of a TSC patient presenting with recurrent severe episodes of retroperitoneal hemorrhage from AMLs successfully managed by angio-embolization.

**Conclusion:**

Transarterial embolization is effective in preventing and controlling hemorrhage in patients with AMLs of the kidney

## Background

Tuberous sclerosis complex (TSC) or Bourneville's disease is a rare genetic disorder, either sporadic or inherited by an autosomal dominant transmission. Mutations in one of two genes, TSC1 and TSC2, have been identified as causes of TSC. Affected patients develop hamartomatous lesions in different organs, most commonly in the brain, eyes, heart, kidney, skin and lungs. Angiomyolipomas (AML) of the kidney are the most common TSC lesion, occurring in 70 to 80% of adults and older children.[1] These lesions can be a bleeding source.

## Case presentation

**Figure 1:**
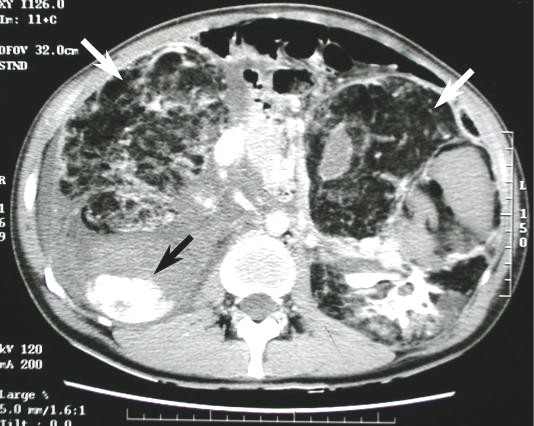
**CT scan showing honeycomb kidneys (white arrows) with contrast blush inside a right retroperitoneal hematoma (black arrow)**.

**Figure 2:**
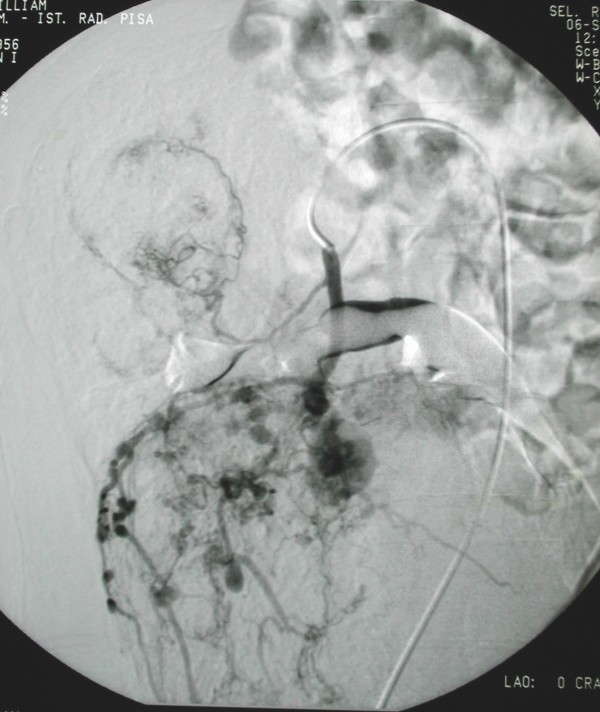
**selective angiography of the right kidney showing giant angiomyolipomas before embolization**.

**Figure 3:**
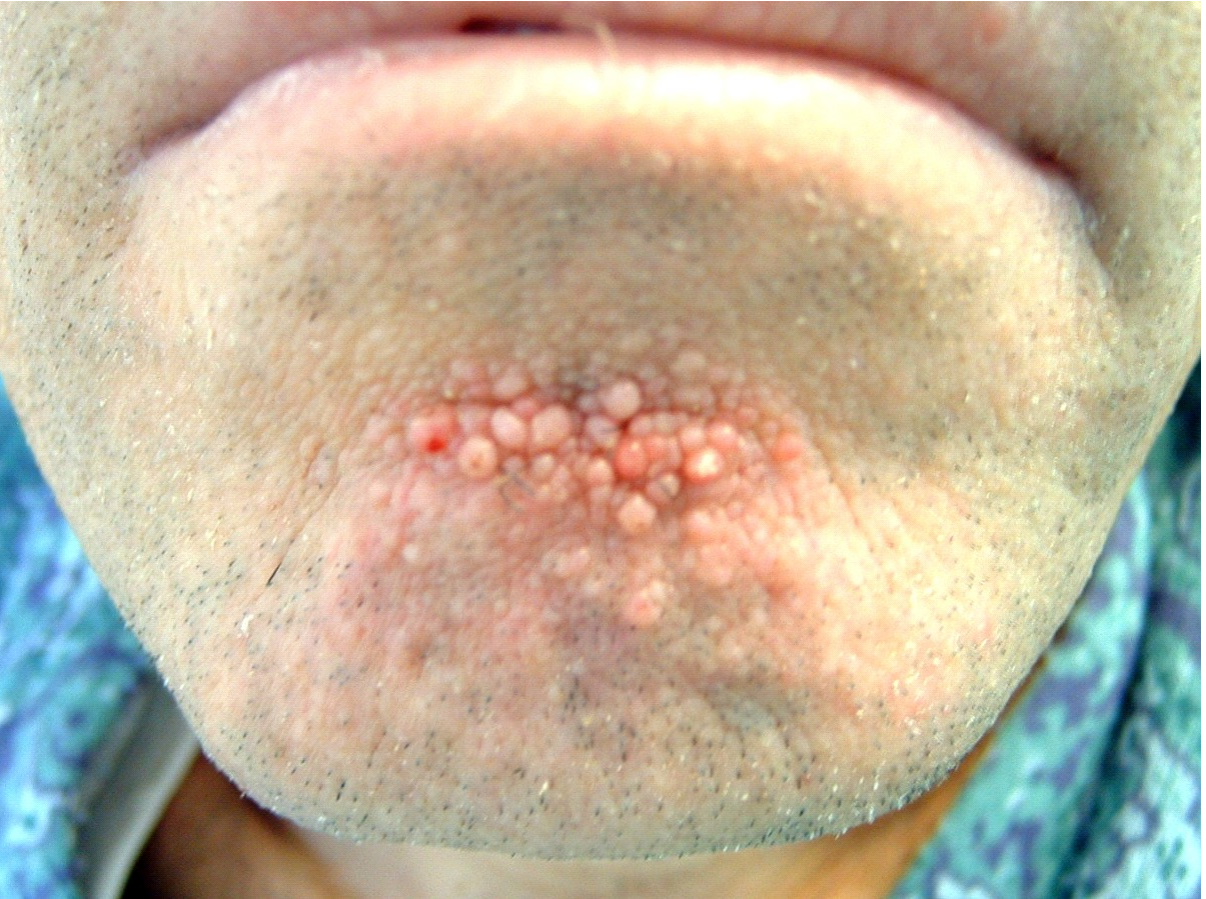
**sebaceous adenomas of the face**.

**Figure 4:**
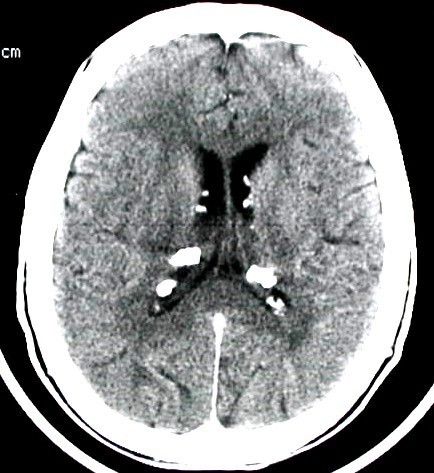
**subependymal calcification as seen at the CT scan**.

**Figure 5:**
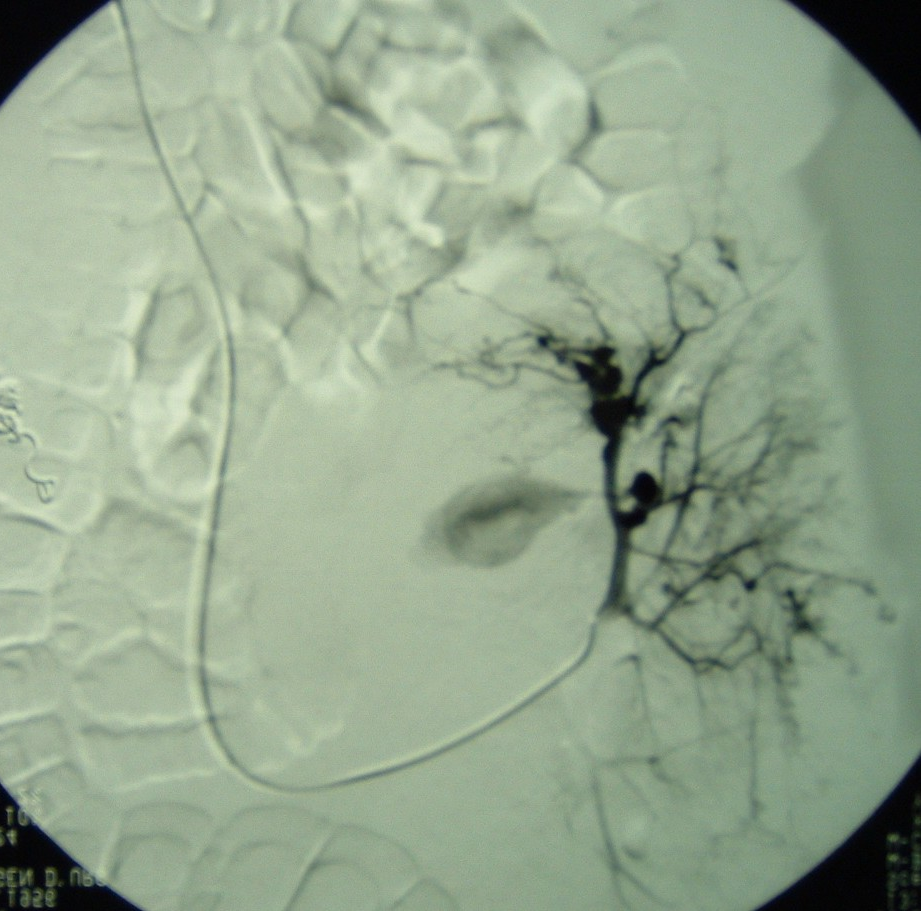
**recurrent episode of retroperitoneal hemorrhage from AML of the left kidney**.

In September 2000, a 48 year-old Caucasian male with sporadic tuberous sclerosis presented to the emergency room with acute right flank pain, large abdominal mass, signs and symptoms of internal bleeding (hypotension, tachycardia and acute anemia). Abdominal CT scan showed bilateral renal giant angiomyolipomatosis with a honey-comb aspect of the kidneys and a right retroperitoneal haematoma. Moreover, an arterial contrast blush was seen within the right retroperitoneal hemorrhagic infarction (Figure 1). The patient underwent angiography (Figure 2), which showed the right kidney to be supplied by two main arteries and the presence of multiple intraparenchymal aneurysms (arrows) in the territory of the lower artery. There was no active bleeding but due to the size of AML and of the aneurysms a selective embolization with metal coils was carried out. Adjunctive features of the syndrome in this patient included sebaceous adenoma of the face (Figure 3), and sub-ependymal calcifications (Figure 4). The course was uneventful and kidney function was maintained. A further episode of massive retroperitoneal bleeding occurred 40 months later. The bleeding source was from a AML lesion of the upper pole of the left kidney (Figure 5). Control was achieved by selective angio-embolization. Since then, there has been no recurrence of retroperitoneal bleeding, but the patient has developed a mild renal failure.

## Discussion

The three major renal manifestations of TSC include AML and less frequently polycystic kidney disease (PKD) and renal cell carcinoma (RCC), arising alone or in combination. Of these, however, only AML are a major criterion for TSC diagnosis because neither the presence of cysts nor RCC is sufficiently specific for TSC. Angiomyolipomas are benign tumors composed of abnormal vessels, immature smooth muscle cells and fat cells. Their incidence in the general population is 1–2%, whereas in patients with TSC the incidence of AML has been estimated at 50–80%. In respect to sporadic AML, patients with TSC significantly have multiple and bilateral AML that tend to grow with time. As AML grow, aneurysms develop inside the mass. Bleeding from ruptured aneurysm increases in frequency when tumors become larger than 4 cm. Giant renal AML in Bourneville's syndrome raise a surgical interest because they are a source of life-threatening hemorrhage. Hemorrhage from AML has been reported among the most common causes of death in patients with TSC.[2] Management of AML greater than 4 cm electively identified by imaging series should be balanced between the need to prevent life-threatening bleeding and the issue of preserve as much functional renal parenchyma as possible. For this purpose a variety of methods have been proposed. These include selective renal angiography and embolization, AML ablation by cryotherapy or radiofrequency and partial nephrectomy.[2,3] In the presence of ongoing bleeding, selective renal embolization is a safe and effective method to achieve hemorrhage control and to reduce the size of AML. This treatment is minimally invasive and preserves renal function. Currently, embolization is proposed as the first choice of intervention for symptomatic AMLs or to prevent hemorrhage when AMLs exceed 4 cm in diameter.[4,5] Patients with TSC have however a 40% recurrence rate of symptomatic AMLs with a median time interval from embolization to recurrence of approximately 79 months.[6] As genes mutations in TSC result in activation of the mammalian target of rapamycin (mTOR), therapy with sirolimus, a suppressor of the mTOR signaling, has been recently implemented in order to reduce the size of AML and to prevent retroperitoneal bleeding.[7] As result of the treatment, AMLs regressed during the therapy but increased again once the therapy was stopped. Some severe adverse events from sirolimus also were reported. Thus, so far, the role of mTOR suppressors in preventing hemorrhage from AML seems to be very promising, but yet, it cannot be considered as standard of care. Most of TSC patients presenting with recurrence and of re-bleeding may be first safety and effectively managed by redo angio-embolization. However some patients with repetitive renal bleeding will eventually require a nephrectomy. For these cases combined nephrectomy and pre-emptive renal transplantation may be the procedure of choice, as it removes the risk of severe bleeding and represents definitive treatment.[8]

## Conclusion

Transarterial embolization is effective in preventing and controlling hemorrhage in patients with AMLs of the kidney. When AMLs are associated to TSC, recurrence of bleeding is not uncommon and redo angio-embolization may be required.

## Consent

Written informed consent was obtained from the patient for publication of this case report and accompanying images. A copy of the written consent is available for review by the Editor-in-Chief of this journal.

## Competing interests

The authors declare that they have no competing interests.

## Authors' contributions

MC and LD carried out the literature research and drafted the manuscript. MP and MC were major contributor to the writing of this case. CV retrieved and processed the images. All authors read and approved the final manuscript.
